# Differences in body composition and metabolic status between white UK and Asian Indian children (EarlyBird 24 and the Pune Maternal Nutrition Study)

**DOI:** 10.1111/j.2047-6310.2012.00063.x

**Published:** 2012-08-31

**Authors:** S Lakshmi, B Metcalf, C Joglekar, C S Yajnik, C H Fall, T J Wilkin

**Affiliations:** 1 Department of Endocrinology & Metabolism, Peninsula Medical SchoolPlymouth Campus, UK; 2 Department of Diabetes, King Edward VI HospitalPune, INDIA; 3 Medical Research CouncilSouthampton, UK

**Keywords:** Body composition, children, metabolic status, transracial

## Abstract

**What is already known about this subject:**

- South Asian children at birth are thinner, but more adipose and more resistant to insulin than White Caucasian children.
- South Asian adults are more adipose and more insulin resistant, but their greater adiposity does not fully explain the difference in insulin resistance.
- South Asian children at 8 y are more insulin resistant than White Caucasian children.

**What this study adds:**

- The BMI of South Asian children at 6 y is distributed normally, while that of White Caucasian children is heavily skewed.
- South Asian children at 6 y are slimmer, and the boys, but not the girls, are more adipose.
- South Asian boys, but not girls are more insulin resistant. Both genders metabolically less healthy, but their adiposity explains only part of the difference.

**Background/Aims:**

The concept of the ‘thin–fat’ Indian baby is well established, but there is little comparative data in older children, and none that examines the metabolic correlates. Accordingly, we investigated the impact of body composition on the metabolic profiles of Asian Indian and white UK children.

**Methods:**

Body mass index (BMI), waist circumference, sum of four skin-folds, % body fat (by dual-energy X-ray absorptiometry), glucose, insulin, insulin resistance (Homeostasis Model Assessment), trigylcerides, cholesterol [total, low-density lipoprotein, high-density lipoprotein {HDL}, total/HDL ratio] and blood pressure (systolic, diastolic and mean arterial) were measured in 262 white Caucasian children from Plymouth, UK (aged 6.9 ± 0.2 years, 57% male), and 626 Indian children from rural villages around Pune, India (aged 6.2 ± 0.1 years, 53% male).

**Results:**

Indian children had a significantly lower BMI (boys: −2.1 kg m^−2^, girls: −3.2 kg m^−2^, both *P* < 0.001), waist circumference (*P* < 0.001) and skin-fold thickness (*P* < 0.001) than white UK children, yet their % body fat was higher (boys +4.5%, *P* < 0.001, girls: +0.5%, *P* = 0.61). Independently of the differences in age and % body fat, the Indian children had higher fasting glucose (boys +0.52 mmol L^−1^, girls +0.39 mmol L^−1^, both *P* < 0.001), higher insulin (boys +1.69, girls +1.87 mU L^−1^, both *P* < 0.01) and were more insulin resistant (boys +0.25, girls +0.28 HOMA-IR units, both *P* < 0.001).

**Conclusions:**

The ‘thin–fat’ phenotype observed in Indian babies is also apparent in pre-pubertal Indian children who have greater adiposity than white UK children despite significantly lower BMIs. Indian children are more insulin resistant than white UK children, even after adjustment for adiposity.

## Introduction

Obesity is a global issue that is reaching epidemic proportions 1. It is the single most important cause of insulin resistance, which is thought to underlie heart disease and diabetes 2. Although widely used for convenience, body mass index (BMI) is an incomplete index of obesity-related health risk because it does not distinguish lean from fat, nor indicate fat distribution. Adiposity, where % body fat is measured indirectly (e.g. bio-impedance) or by criterion methods [e.g. dual-energy X-ray absorptiometry {DEXA}], is a more precise measure of fatness.

Indians tend to have a higher proportion of body fat than white Caucasians, and more of it is distributed within the abdominal cavity 3. Body composition, and fat distribution in particular, is important because of its metabolic implications. For reasons that are not fully understood, fat deposited within the abdominal cavity is associated with insulin resistance, while subcutaneous fat is not. It may be that the inflammatory profile of intra-abdominal adipocyte secretions is important (the adipokine hypothesis) or that visceral fat is a marker for the deposition of ectopic fat in insulin-sensitive liver and muscle [the ‘overflow hypothesis’ 4]. It has been known for some time that Indians, even from infancy, are more insulin resistant than white Caucasians 5,6, and a proportionately greater distribution of fat within the abdomen is one possible reason.

While it can be assumed that such differences reflect evolutionary adaptations to different environments, problems can arise when individuals, shaped by one environment, move to another 7. Rural India is in a state of flux, such that millions are migrating to an urbanized lifestyle of limited physical activity and calorie-dense nutrition. The result is a rising level of urban obesity, diabetes and heart disease 8. The greater adiposity (% body fat) of Indian compared with white Caucasian populations has been reported in adults, adolescents and even infants 9–11.

The concept of the ‘thin–fat’ Indian baby is well established 12, but there is little comparative information in older children. This study aims to investigate whether the ‘thin/fat’ Indian still exists by age 6–7 years by comparing the BMI and body composition of Asian Indian children with white Caucasian UK children of similar age. Importantly, it also aims to test to what extent any observed metabolic differences could be explained by differences in body composition.

## Methods

We compared the anthropometry, body composition and metabolic status of children from two large cohorts: one from the city of Plymouth, UK, and the other from rural villages surrounding Pune in Southern India. The study was cross-sectional.

### The EarlyBird diabetes study

The EarlyBird diabetes study (EBDS) has been monitoring a 1995/1996 birth cohort of 307 healthy children from the city of Plymouth in the South of England since the age of 5 years. The recruitment process and conduct of the study have been described in detail elsewhere 13. The children were selected randomly across the socioeconomic range from 53 primary schools and are reviewed annually. This study relates to 262 children (149 boys) with complete data sets, examined at mean age 6.9 ± 0.2 years. Ethical approval was granted in 1999.

### The Pune maternal nutrition study

The Pune maternal nutrition study (PMNS) was established in 1993 and has monitored from conception a 1995/1996 birth cohort raised in rural villages around Pune in Southern India 14. All married women of reproductive age living in six villages (*n* = 2675) were recruited (from June 1994 to April 1996), and of these, 1102 became pregnant, and 762 delivered live babies. The data set analysed here relates to the 626 children (330 boys) with complete data sets at a mean age of 6.2 ± 0.1 years. The study was designed to examine the relationship between a mother's size, body composition, energy and protein intakes, and micronutrient status on foetal and subsequent growth.

### Anthropometry and body composition

Height (stadiometer) and weight (calibrated scales) were measured in both cohorts by trained research nurses, and body composition by the same model of DEXA (Lunar DPX, Madison, WI, USA) using the same software (version 4) and the same model of calibration block supplied by the manufacturer. The software reports body composition as the mass and proportion (%) of fat, lean (muscle and organs), bone and water. Calipers (EBDS: Holtain Ltd, Crosswell, Crymych, Dyfed; PMNS: Harpenden, CMS Instruments Ltd, London) were used in both cohorts to measure the sum of four skin-fold thicknesses (biceps, triceps, subscapular and suprailiac), an index of subcutaneous fatness.

### Metabolic markers

All blood analyses were performed on fasting samples in both populations.

#### The EarlyBird diabetes study

*Insulin* was measured by immunometric assay on a DPC Immulite analyzer (Diagnostic Products Corporation, Los Angeles, CA, USA). Insulin cross-reactivity with proinsulin was less than 1%. *Glucose*, *cholesterol*, *high-density lipoprotein (HDL) cholesterol* and *triglycerides* were measured on a Cobas Integra 700 analyzer (Roche Diagnostics, Lewes, East Sussex, UK). *Blood pressure* was taken by semi-automated sphygmomanometer (Welch-Allyn, Beaverton, OR, USA) and the mean of the second and third of three recordings used in the analysis.

#### Pune maternal nutrition study

Plasma glucose and serum cholesterol, HDL cholesterol, and triglyceride concentrations were measured in Pune, India, using standard enzymatic methods (Spectrum; Abbott, Irving, TX, USA). Between-batch coefficients of variation for all the assays were <3% in the normal range. Insulin was measured using a two-site immunoenzymometric assay (Medgenix, Fleurus, Belgium), which did not cross-react with proinsulin. Between-batch coefficients of variation were 11% at 24 pmol L^−1^, 8% at 6.0 pmol L^−1^ and 4.8% at 477 pmol L^−1^. Systolic and diastolic blood pressures were measured after 5 min resting quietly, using a digital semi-automated device (Dinamap; Criticon, Tampa, FL, USA).

Insulin resistance was modelled in both populations from fasting insulin and glucose using Homeostasis Model Assessment (HOMA2-IR) programme 15, and a measure of mean arterial blood pressure calculated – (systolic blood pressure [SBP] + 2 × diastolic blood pressure [DBP])/3.

### Statistical analysis

Measures of body dimensions, body composition and metabolic markers are expressed as medians and inter-quartile ranges (from 25th to 75th centiles) for both sexes separately and for each country. The Mann– Whitney *U*-test (non-parametric) was used to detect between-country differences. Multiple linear regression modelling was then used to determine the between-country differences for each metabolic marker after controlling for between-country differences in age and total fat %. Further adjustment for skin-fold thickness was not made as the strength of the association between skin-fold thickness and some metabolic markers differed by country (‘country × sum of four skin-folds’ interaction terms, *P* < 0.05). The residuals obtained from modelling insulin and insulin resistance violated the assumption of normality (they were positively skewed) and of constant variance (they were heteroscedastic) and were therefore analysed in as their natural logarithm. The resulting coefficients produced from the analysis of logged data represent the percentage difference, rather than absolute difference, in the dependent variable between the two countries. All analysis was carried out using SPSS version 17.0 for Windows (SPSS, Chicago, IL, USA).

## Results

### Observed differences

#### Body size, mass and fat

The UK children were approximately 9 months older, 11 cm taller and 7 kg heavier than the Indian children (Table 1). UK boys had a significantly greater BMI (+2.1 kg m^−2^), waist circumference (+3.2 cm) and sum of four skin-folds (+1.3 cm) than Indian boys, yet their % body fat was significantly lower (−4.5%). UK girls also had a significantly greater BMI (+3.2 kg m^−2^), waist circumference (+3.8 cm) and sum of four skin-folds (+1.8 cm) than Indian girls, but their % body fat was similar (−0.5%).

**Table 1:** Anthropometric and body composition measurements in children from the UK (EBDS cohort) and India (PMNS cohort)

Measure	Sex	UK	India	*P*
*N*	Boys	149	330	–
Girls	113	296	–
Age (year)	Boys	6.89 (6.72 to 7.03)	6.13 (6.05 to 6.24)	<0.001
Girls	6.85 (6.71 to 6.98)	6.16 (6.07 to 6.25)	<0.001
Height (cm)	Boys	122 (118 to 126)	110 (107 to 113)	<0.001
Girls	121 (118 to 126)	110 (107 to 113)	<0.001
Weight (kg)	Boys	22.7 (20.4 to 25.1)	16.4 (15.4 to 17.7)	<0.001
Girls	23.0 (20.7 to 26.9)	15.9 (14.8 to 17.2)	<0.001
BMI (kg m^−2^)	Boys	15.7 (14.8 to 16.9)	13.6 (12.9 to 14.1)	<0.001
Girls	16.3 (15.2 to 18.1)	13.1 (12.5 to 13.8)	<0.001
Total body fat (%)	Boys	13.1 (9.9 to 17.4)	17.6 (14.1 to 20.5)	<0.001
Girls	19.9 (14.4 to 26.3)	20.4 (17.5 to 24.0)	0.61
Waist circumference (cm)	Boys	53.5 (51.8 to 55.8)	50.3 (48.6 to 52.2)	<0.001
Girls	53.8 (51.5 to 58.3)	50.0 (48.3 to 51.8)	<0.001
Sum of four skin-folds (cm)	Boys	3.24 (2.78 to 4.01)	1.95 (1.73 to 2.19)	<0.001
Girls	4.02 (3.22 to 5.49)	2.18 (1.95 to 2.49)	<0.001

Values are median (from 25th to 75th centiles).

*P* values derived from Mann–Whitney *U*-test.

BMI, body mass index; EBDS, EarlyBird diabetes study; PMNS, Pune maternal nutrition study.

**Figure 1:**
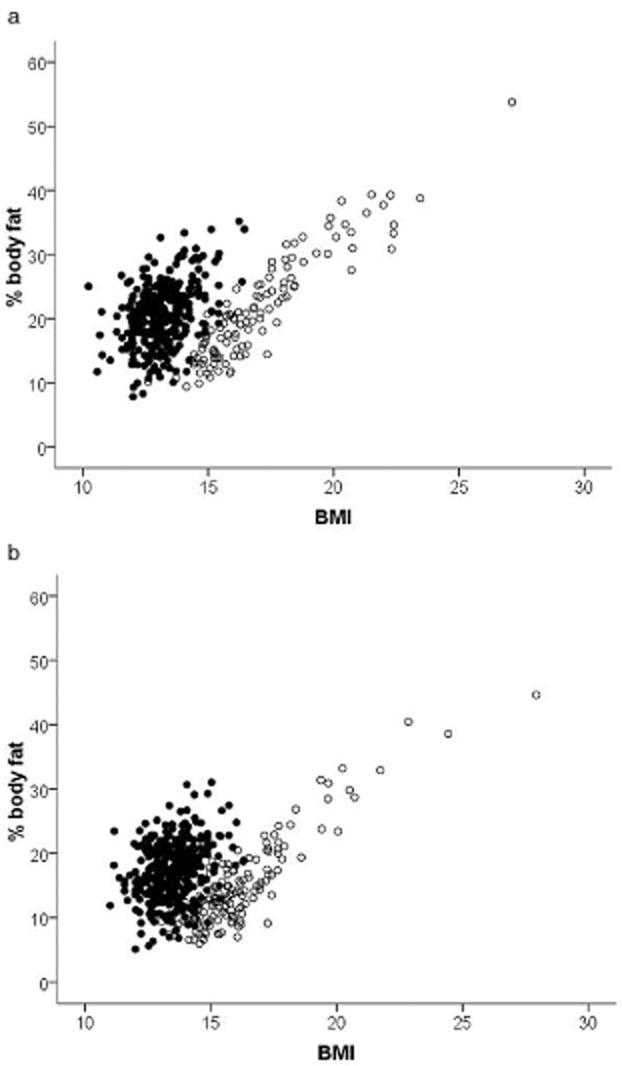
The distribution of, and the relationship between, body mass index (BMI) and % body fat in white UK (open circle) and Asian Indian (closed circle) children (a – boys, b – girls).

Figure 1 shows that the distribution of BMI and % fat are both skewed in the UK children, but Gaussian in the Indian children. The figure again illustrates the difference in correspondence between BMI and % fat in the two races.

#### Metabolic markers

In both sexes, glucose was higher in the Indian children compared with the UK children (Table 2). Indian children also tended to have higher insulin levels and HOMA-IR, although not significantly so in the girls. Again, triglycerides and the cholesterol/HDL ratio were higher in the Indian children, while systolic, diastolic and mean arterial blood pressures were significantly lower. Racial differences in the relationship between % fat and insulin resistance are explored in Figure S1.

**Table 2:** Metabolic markers in children from the UK (EBDS cohort) and India (PMNS cohort)

Marker	Sex	UK	India	*P*
*N*	Boys	149	330	–
Girls	113	296	–
Glucose (mmol L^−1^)	Boys	4.65 (4.40 to 4.90)	5.05 (4.72 to 5.33)	<0.001
Girls	4.60 (4.30 to 4.80)	4.83 (4.56 to 5.17)	<0.001
Insulin (mU L^−1^)	Boys	2.0 (1.5 to 3.7)	3.0 (1.6 to 4.6)	0.004
Girls	2.9 (2.0 to 4.1)	3.4 (1.9 to 5.2)	0.11
Insulin resistance (HOMA-IR)	Boys	0.29 (0.22 to 0.53)	0.46 (0.23 to 0.69)	0.001
Girls	0.42 (0.28 to 0.59)	0.50 (0.28 to 0.76)	0.050
Triglyceride (mmol L^−1^)	Boys	0.52 (0.39 to 0.69)	0.66 (0.55 to 0.80)	<0.001
Girls	0.60 (0.48 to 0.76)	0.64 (0.54 to 0.81)	0.029
Cholesterol (mmol L^−1^)	Boys	4.1 (3.7 to 4.6)	3.3 (2.9 to 3.7)	<0.001
Girls	4.2 (3.8 to 4.7)	3.3 (2.9 to 3.7)	<0.001
LDL-C (mmol L^−1^)	Boys	2.3 (1.9 to 2.7)	1.8 (1.5 to 2.2)	<0.001
Girls	2.4 (2.0 to 2.8)	1.9 (1.5 to 2.3)	<0.001
HDL-C (mmol L^−1^)	Boys	1.6 (1.4 to 1.9)	1.1 (1.0 to 1.3)	<0.001
Girls	1.5 (1.4 to 1.8)	1.1 (0.9 to 1.2)	<0.001
Cholesterol/HDL (ratio)	Boys	2.6 (2.2 to 2.9)	3.0 (2.6 to 3.5)	<0.001
Girls	2.8 (2.4 to 3.2)	3.0 (2.7 to 3.7)	<0.001
SBP (mm Hg)	Boys	100 (94 to 104)	92 (85 to 98)	<0.001
Girls	96 (92 to 101)	88 (81 to 97)	<0.001
DBP (mm Hg)	Boys	61 (57 to 65)	54 (48 to 60)	<0.001
Girls	60 (58 to 65)	54 (46 to 59)	<0.001
MAP (mm Hg)	Boys	74 (70 to 77)	67 (61 to 72)	<0.001
Girls	72 (70 to 77)	65 (58 to 71)	<0.001

Values are median (from 25th to 75th centiles).

*P* values derived from Mann–Whitney *U*-test.

DBP, diastolic blood pressure; EBDS, EarlyBird diabetes study; HDL-C, high density lipoprotein-cholesterol; HOMA-IR, homeostatic model assessment-insulin resistance; LDL-C, low density lipoprotein-cholesterol; MAP, mean arterial pressure; PMNS, Pune maternal nutrition study; SBP, systolic blood pressure.

### Adjusted differences

Differences in age and % fat were unable to explain the higher levels of glucose, insulin, insulin resistance and cholesterol/HDL ratio among the Indian children, nor the higher blood pressure levels in the UK children (Table 3). Only the differences in triglycerides became non-significant once adjusted for age and % fat.

**Table 3:** Differences in the metabolic markers between UK children (EBDS cohort) and Indian children (PMNS cohort) adjusted variously for age and body composition

	Boys		Girls	
UK – India	*P*	UK – India	*P*
	Beta coefficient (95% CI)		Beta coefficient (95% CI)	
Glucose (mmol L^−1^)				
Model 1	−0.52 (−0.72 to −0.32)	<0.001	−0.39 (−0.61 to −0.17)	<0.001
Model 2	−0.50 (−0.70 to −0.30)	<0.001	−0.38 (−0.60 to −0.16)	<0.001
Insulin* (%)				
Model 1	−43 (−25 to −57)	<0.001	−42 (−21 to −57)	<0.01
Model 2	−36 (−15 to −52)	<0.001	−39 (−18 to −54)	<0.01
Insulin resistance* (%)				
Model 1	−45 (−27 to −58)	<0.001	−44 (−24 to −58)	<0.001
Model 2	−38 (−17 to −53)	<0.001	−40 (−21 to −55)	<0.001
Triglycerides (mmol L^−1^)				
Model 1	−0.10 (−0.20 to 0.00)	0.04	0.03 (−0.09 to 0.15)	0.58
Model 2	−0.08 (−0.18 to 0.02)	0.13	0.04 (−0.08 to 0.16)	0.51
Total cholesterol (mmol L^−1^)				
Model 1	0.73 (0.47 to 0.99)	<0.001	0.90 (0.60 to 1.20)	<0.001
Model 2	0.78 (0.52 to 1.04)	<0.001	0.92 (0.62 to 1.22)	<0.001
LDL cholesterol (mmol L^−1^)				
Model 1	0.35 (0.11 to 0.59)	<0.01	0.41 (0.15 to 0.67)	<0.01
Model 2	0.39 (0.15 to 0.63)	<0.01	0.44 (0.18 to 0.70)	<0.01
HDL cholesterol (mmol L^−1^)				
Model 1	0.41 (0.29 to 0.53)	<0.001	0.48 (0.36 to 0.6)	<0.001
Model 2	0.41 (0.29 to 0.53)	<0.001	0.48 (0.36 to 0.6)	<0.001
Total/HDL cholesterol (ratio)				
Model 1	−0.44 (−0.72 to −0.16)	<0.01	−0.48 (−0.80 to −0.16)	<0.01
Model 2	−0.40 (−0.68 to −0.12)	<0.01	−0.46 (−0.78 to −0.14)	<0.01
Systolic BP (mm Hg)				
Model 1	8.5 (4.1 to 12.9)	<0.001	12.1 (6.5 to 17.7)	<0.001
Model 2	8.3 (3.9 to 12.7)	<0.001	12.3 (6.7 to 17.9)	<0.001
Diastolic BP (mm Hg)				
Model 1	7.1 (3.5 to 10.7)	<0.001	12.4 (8.2 to 16.6)	<0.001
Model 2	7.5 (3.7 to 11.3)	<0.001	12.5 (8.3 to 16.7)	<0.001
Mean arterial BP (mm Hg)				
Model 1	7.6 (4.0 to 11.2)	<0.001	12.3 (7.9 to 16.7)	<0.001
Model 2	7.8 (4.2 to 11.4)	<0.001	12.4 (8.0 to 16.8)	<0.001

Model 1 is adjusted for age, model 2 is adjusted for age and % fat.

* Analysis carried out where the outcome data was log-transformed; hence, the coefficients represent the percentage difference in the dependent variable between the two countries. All other coefficients represent the absolute difference derived from the analysis of raw data.

BP, blood pressure; CI, confidence interval; EBDS, EarlyBird diabetes study; HDL, high-density lipoprotein; LDL, low-density lipoprotein; PMNS, Pune maternal nutrition study.

There were racial differences in the relationship between skin-fold thickness and both fasting insulin and HOMA-IR independent of % fat. For example, a thicker skin-fold thickness relative to % fat was associated with higher levels of insulin resistance in the white UK children (although not significantly so, boys *r* = 0.14, girls *r* = 0.11, both *P* > 0.1) but lower insulin resistance in the Indian children (boys *r* = −0.21 *P* < 0.001, girls *r* = −0.15 *P* = 0.03). This ‘country × sum of four skin-folds’ interaction remained, even when the analysis was carried out on a subsample of UK children that had comparable fatness levels to the Indian children (data not shown).

## Discussion

Comparisons of metabolic markers in relation to body composition have not been made before in white Caucasian and Indian children using criterion methods, and the study reported here draws strength from its large numbers and uniformity of age. Our findings point to some fundamental differences between the races. The Indian child has a lower BMI and narrower waist circumference but tends to have greater adiposity (higher % body fat) and to have a generally less favourable metabolic profile. However, of the several metabolic variables tested, only the higher triglycerides could be explained by differences in body composition. Insulin resistance appears to be intrinsically higher in the Indian child and may be related to the distribution of body fat.

The experience of others has been variable. Whincup and colleagues, comparing white Caucasian with, this time British, South-Asian children, adjusted for differences for fatness by weight-for-height rather than % body fat, but they were again unable to explain the differences found in insulin resistance 16. Ehtisham and colleagues, on the other hand, reported that body fat measured by DEXA accounted entirely for the higher insulin resistance (lower insulin sensitivity) among British Indian adolescents aged 14–17 years 10. Differences in age and/or environment might explain the discrepancy with our findings, although environment seems less likely given the strong association between urban setting and insulin resistance in Indian men 17. Yajnik and colleagues found insulin resistance to be 84% higher in urban than in rural Indian men, but it was still left with 32% of the difference after accounting for adiposity.

The observations that Indian children have less subcutaneous fat but more fat overall than white UK children infer that a substantial proportion of body fat in Indian children is located outside the subcutaneous compartment, most probably within the abdominal cavity 18. Indeed, it has shown recently by magnetic resonance imaging scanning that Indian babies have significantly more intra-abdominal fat (by ∼2 standard deviation) than white babies 19. Furthermore, we found racial differences in the relationship between skin-fold thickness and both fasting insulin and HOMA-IR independent of % fat such that a thinner skin-fold relative to % body fat was detrimental to insulin and insulin resistance in Indian children, but unrelated in UK children. The metabolic disadvantage of relatively low subcutaneous fat seems likely to reflect a correspondingly greater intra-abdominal fat mass, although we had no direct measure for it. Intra-abdominal fat is associated with loss of insulin sensitivity, and such differences in fat distribution may explain why Indian children are more insulin resistant 20.

The diet of rural Indian children is largely unprocessed and likely to contain less energy than that of UK children. Under such circumstances, body mass and adiposity appear to be normally distributed, in distinction to the skewed distribution among urbanized UK children of 6 years old, which most probably reflects a selective, genetically determined, response to mounting obesogenic pressures 21. It is not clear whether skewing necessarily points to a metabolically unhealthy society 22, for BMI is not a measure of body composition, but the difference is striking.

This study has strengths and limitations. It is larger than many such comparisons, involves cohorts of similar age and uses the criterion method of DEXA to assess body composition and the same model of DEXA scanner in each centre. The metabolic assays were not cross-validated but were subject to local standard quality-control procedures. Although ideal, cross-validation is seldom available to international comparisons of this kind. The aim of this study was to compare pre-industrialized Indian children with urbanized Caucasian children. Future studies might usefully complete the circle by including the body composition and metabolic profiles of rural Indian children from the same region who have migrated to the urbanized West.

In conclusion, differences in the body composition of Indian and white Caucasian children are unable to fully explain differences in their metabolic status. The incidence of diabetes in such populations is much greater, and a precise explanation for the difference is needed.

## Conflict of interest statement

No conflicts of interest was declared.

## Supporting Information

Figure S1 The relationship between % body fat and insulin resistance in white UK (open circle) and Asian Indian (closed circle) children (A – boys, B – girls)
