# Supplementary material for: Differences in body composition and metabolic status between white UK and Asian Indian children (EarlyBird 24 and the Pune Maternal Nutrition Study)
Source: Pediatr Obes. 2012 Aug 31;7(5):347–54. doi: 10.1111/j.2047-6310.2012.00063.x (PMC3541477; doi:10.1111/j.2047-6310.2012.00063.x)
Supplement: Figure S1 — The relationship between % body fat and insulin resistance in white UK (open circle) and Asian Indian (closed circle) children (A – boys, B – girls) [file ijpo0007-0347-sd1.doc]

Figure 2: The relationship between %body fat and insulin resistance in White UK (open circle) and Asian Indian (closed circle) children. (A - boys, B - girls)

A

B
